# Supplementary figures and images for: Baseline Gut Metagenomic Functional Gene Signature Associated with Variable Weight Loss Responses following a Healthy Lifestyle Intervention in Humans
Source: mSystems. 2021 Sep 14;6(5):e00964-21. doi: 10.1128/mSystems.00964-21 (PMC8547453; doi:10.1128/mSystems.00964-21)

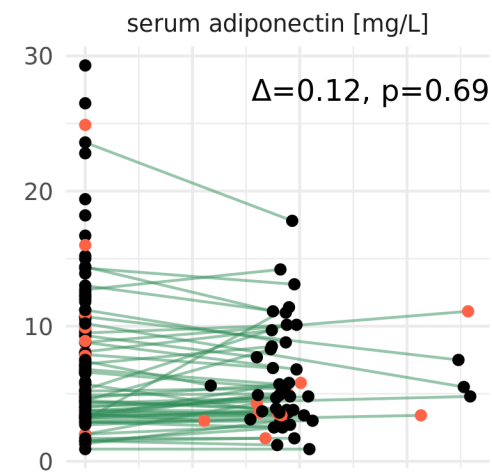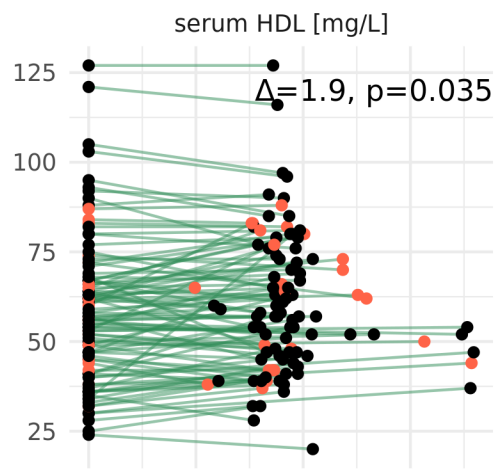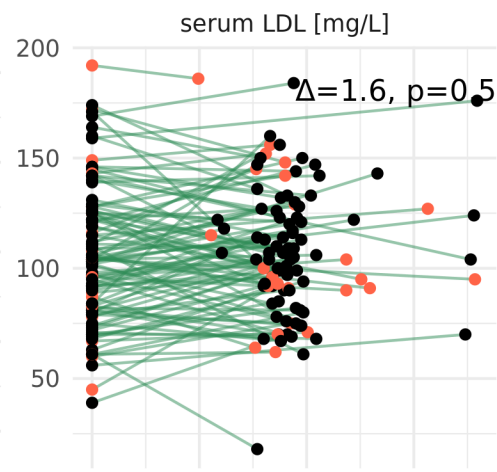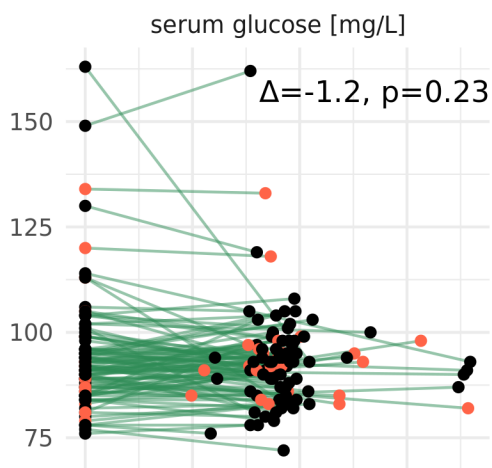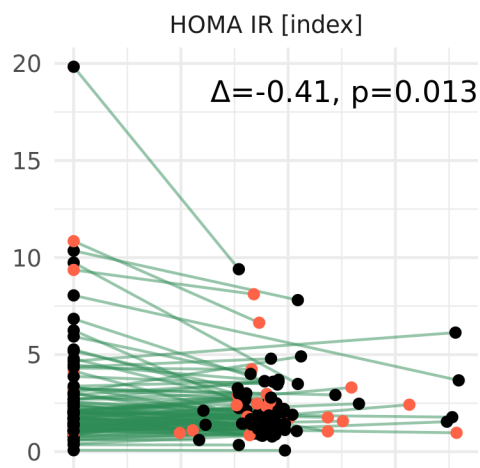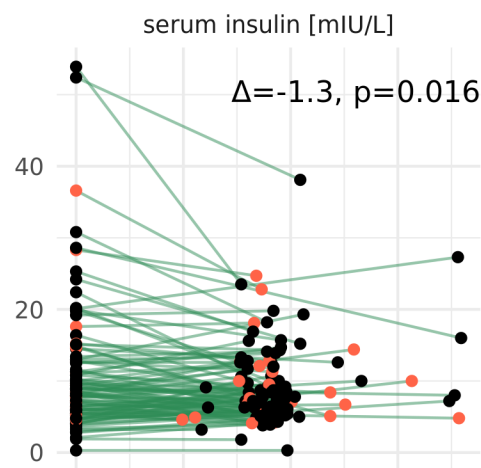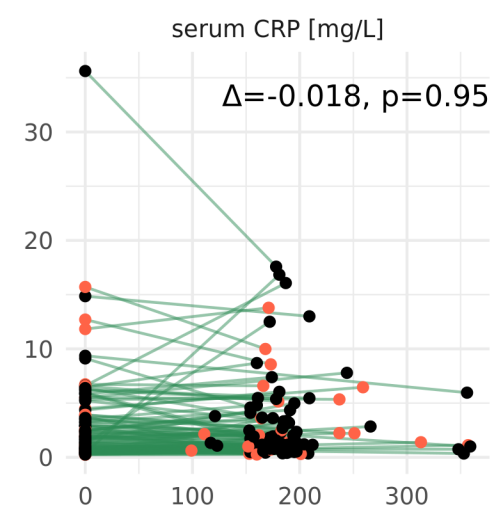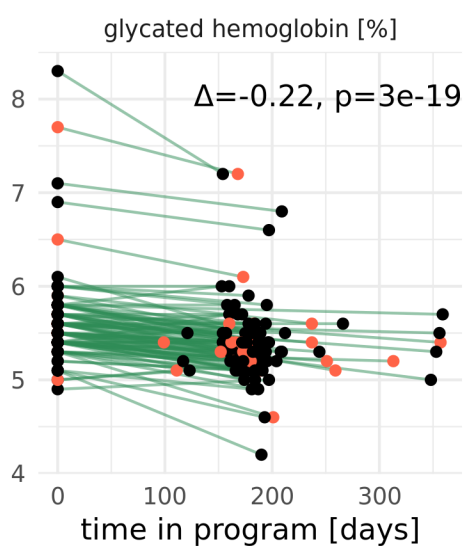

time in program [days]

Supplement: FIG S1 [file msystems.00964-21-sf001.pdf]

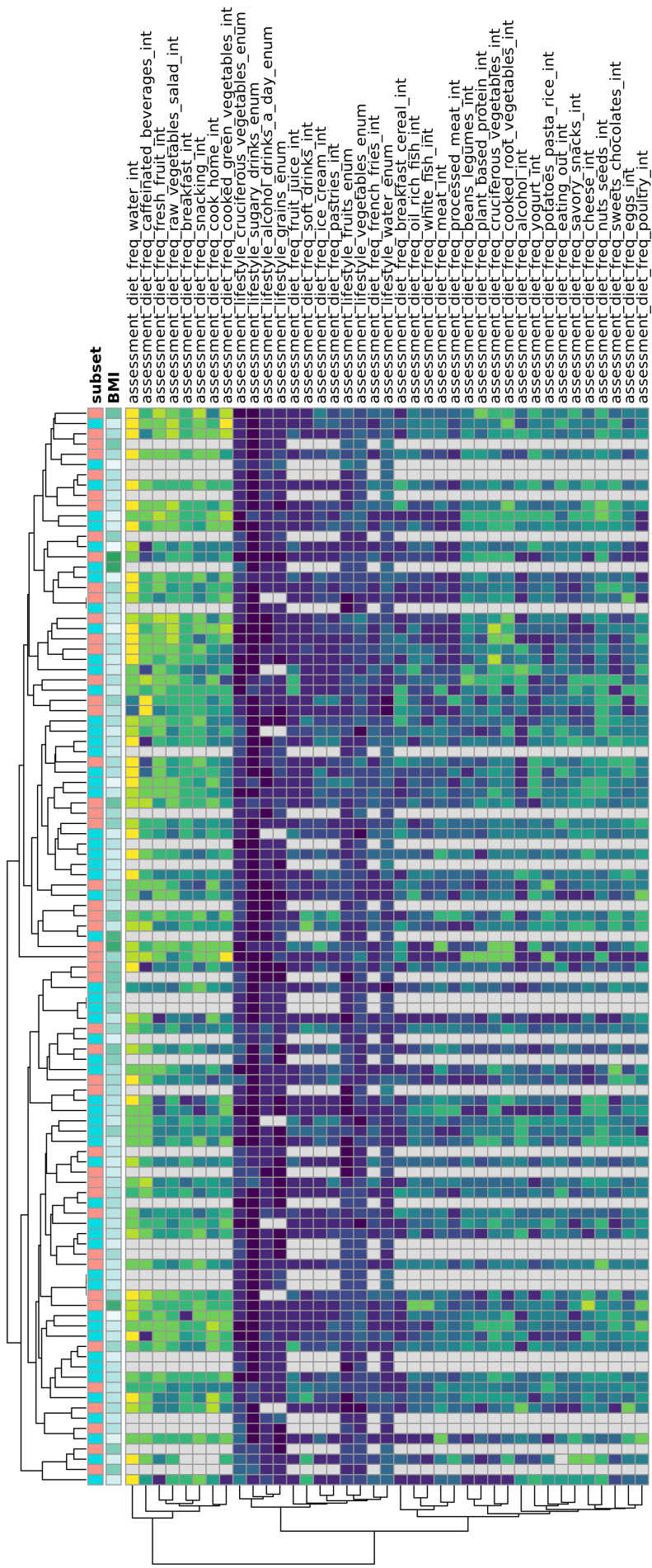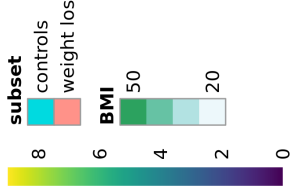

Supplement: FIG S2 [file msystems.00964-21-sf002.pdf]

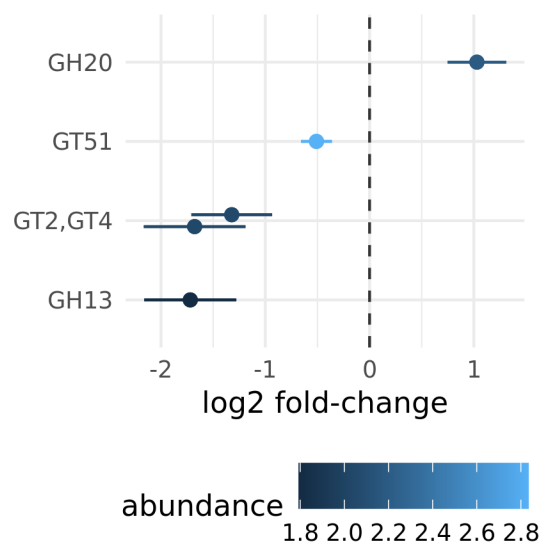

Supplement: FIG S3 [file msystems.00964-21-sf003.pdf]
